# Morphological asymmetry, sex and dominant somatotype among Polish youth

**DOI:** 10.1371/journal.pone.0238706

**Published:** 2020-09-11

**Authors:** Magdalena Krzykała, Małgorzata Karpowicz, Ryszard Strzelczyk, Beata Pluta, Karolina Podciechowska, Krzysztof Karpowicz

**Affiliations:** 1 Department of Recreation, Poznan University of Physical Education, Poznan, Poland; 2 Department of Theory and Methodology of Team Sport Games, Poznan University of Physical Education, Poznan, Poland; 3 Department of Theory of Sport, Poznan University of Physical Education, Poznan, Poland; University of Mississippi, UNITED STATES

## Abstract

The aim of the present study was to determine the level of morphological asymmetry among the general population of Polish youth as it correlates to sex and body type. The anthropometric characteristics of a group of 618 Polish youths (354 males aged 19.5 ± 1.2 years and 264 females aged 19.2 ± 1.2 years) were evaluated to assess their somatotypes using the Heath–Carter method. Body composition was calculated using the bioelectrical impedance method, and the absolute asymmetry index was used for comparisons between the right and left sides of the body. Significant differentiation was observed between all morphological characteristics (≤ 0.0001) and two body types (≤ 0.0001) among sexes. Females and males largely exhibited endomorphic and mesomorphic somatotypes, respectively. The findings demonstrated that dominant somatotype and sex both affect the level of fat mass asymmetry in the arms and legs. Furthermore, significant variations in the levels of asymmetry between sexes were observed in fat mass in both the arms and legs, with greater variation observed in the arms. In the case of muscle mass, no great differences were observed between men, women, or body types. This study provides new data on the morphological asymmetry of given body composition according to somatotype and sex. This study has potential medical implications, given that a large degree of inter-limb imbalance could be shown to have a negative effect on health; the findings of the present study would therefore be important for arriving at an understanding of how to prevent such imbalances, or to mitigate their negative effects.

## Introduction

The human body varies in size according to many factors (e.g., sex, age, climate and physical activity level) and can be described based on its size (e.g., body height and mass), proportion, somatotype and composition (e.g., muscle mass and body fat) [1]. Those differences can also relate to levels of asymmetry in morphological traits. The contralateral limb is often used as an intra-subject control to assess bone size, shape, density or structural properties—or changes to such as a result of infection, tumor, fracture or asymmetric loading stresses [2]. Asymmetry may be more prevalent in specific body segments; examples include the length of upper limb favoring the right side, and asymmetry in the lower limbs favoring the left side [3]. Minor limb asymmetry is common and more noticeable in the upper limbs than in the lower limbs [4, 5]. Right-handed individuals have larger dimensions on their right side, whereas left-handed people have larger dimensions on their left side [6].

Comparisons of upper and lower limbs reveal that the upper extremities are more predisposed to greater asymmetry in muscle mass, body fat or bone mineral density, and such asymmetry increases with work experience. This greater predisposition to asymmetry is connected with those extremities’ greater involvement during unilateral activity—for example, in some occupation involving varied force, such as tailoring or carpentry [6–8]. If one side of the body is stronger than the other, it is very likely that this side is also slightly larger. Furthermore, numerous studies have indicated asymmetric distributions of muscle, bone or fat mass among the limbs of athletes [9–11]. In many sports disciplines where unilateral actions are common such as tennis, field hockey or fencing higher levels of asymmetry exist and could result in athletes developing asymmetric neuromuscular adaptations of the lower extremities [12]. This can be connected to strength and power, with a weaker lower limb being unable to produce or absorb the same amount of force as the stronger limb, which often leads to injuries or poor performance, depending on the level of asymmetry [13]. Bell et al. [14] reported that lean mass asymmetry in the lower extremities is at least partially responsible for asymmetry in power and force. It is suggested, then, that anthropometric data should be gathered if an asymmetry is detected in the functional test, because of the potentially increased risk of injury [15].

Asymmetry would seem to be connected with health and disease, but such studies have not been well supported. There has been some indication that skeletal asymmetries may be related to health issues; for example, pelvic asymmetry can lead to lower back pain, and vertebral asymmetry has been linked to scoliosis [16, 17]. It could also lead to injuries, so the detected asymmetries should be systematically monitored and compensated for if necessary—not only in people who engage in unilateral activities, but also in the general population. Findings regarding asymmetry and physical health are, however, inconsistent. Moreover, there has been extensive research on asymmetry and the performance of athletes. We also know that in older people, asymmetry in lean mass of lower-extremity is associated with functional impairment and mobility, (e.g., gait ability) [18]. Notably, as yet little is known about the asymmetry levels of young people not engaged in regular physical activity [19, 20].

At issue is the question of what constitutes a "normal," optimal level of asymmetry, and at what point a deviation from this range can lead to health problems and increased risk of injury [21] or—in the case of athletes—can negatively affect sports performance [14]. This also applies to morphological and functional asymmetry. The complexity of this problem is still difficult to define. Knowledge of the asymmetry level may be relevant, especially for control and prevention, but also could influence the efficiency of rehabilitation (treatment) programs by decreasing rehabilitation time after injury and allowing an earlier return to physical activity—which may be of particular concern in the case of athletes. The present study, however, doesn’t answer the question of what level of morphological asymmetry needs to be corrected through symmetrical exercises or in rehabilitation programs; rather, it indicates the need to pay attention to this phenomenon among young people.

There is little information on gender differences in morphological asymmetry. In general, regional body weight distribution differs considerably between the sexes [22]. Some results reveal that little gender diversity exists in lower limb length, whereas in the maximum lengths of upper limbs, females were more asymmetrical and right-biased, compared to men [3].

It has been shown that asymmetry also has a positive correlation with body dimension, with larger people (taller and heavier) being more symmetrical. For example, fluctuating asymmetry in arm circumference, as well as in the triceps skinfold, increases with increased body weight, while directional asymmetry in arm and estimated mid-arm muscle circumferences decreases significantly with increases in body weight [23]. The findings obtained by Ozener and Ertugrul [24] showed that increasing the degree of asymmetry of selected morphological traits was associated with decreasing average height in young males (the shorter the young male, the greater the asymmetry level). Also, an association between increased asymmetry and decreased body size was observed in Kirchengast’s study [20]. Another result showed that body mass index (BMI) does not directly reflect the relationship between body composition and asymmetry [6].

To our knowledge, there are no studies that analyze the connection between somatotype, sex and morphological asymmetry level. Our prediction was that inter-limb differences in morphological traits increase with a higher proportion of that traits in body weight. Therefore, the aim of the present study was to determine the level of morphological asymmetry of the general population of Polish youth with regard to sex and body type.

## Materials and methods

This research project was approved by the Human Ethics Research Committee of Adam Mickiewicz Medical University in Poznan, Poland (908/16). All participants received a clear explanation of the study, including the risks and benefits of participation, and provided informed written consent.

The present study was based on the findings of research conducted from 2016–2018 among first-year students at Poznan University of Physical Education. The sample included 354 males aged 19.5 ± 1.2 years and 264 females aged 19.2 ± 1.2 years.

### Procedures

The protocol included anthropometry and body composition assessments using bioelectric impedance analysis (BIA). All measurements were evaluated by trained personnel following standardized procedures. Physical measurements were taken by a highly trained and experienced observer following the recommendations of the International Society for the Advancement of Kinanthropometry [25], while the test date was adapted to academic schedules. Asymmetry of body fat and muscle mass (in percentage) on left and right body segments (upper and lower limbs) measured with BIA was calculated as a percentage of absolute asymmetry following the standard procedure [3]. All measurements were made indoors, where the temperature was held constant at 22 degrees Celsius.

### Anthropometry and body composition

Standing height was measured to the nearest mm using a stadiometer (GPM, Swiss). Body mass (barefoot and wearing light clothing) was measured to within 0.1 kg using a portable digital scale (Wagi Wielkopolska^®^, Poland).

Four skinfold thicknesses were measured from the right side of the body to the nearest 0.1 mm, using a Harpenden caliper (Baty International, UK). The pressure applied by the grasp of the tool was tested in accordance with the manufacturer’s specifications and was constant at 10 g/cm^2^. The following sites were measured: triceps—midway between the acromial and olecranon processes in the midline of the back of the arm; suprailiac—immediately superior to the iliac crest in the midaxillary line; subscapular—the undermost tip of the inferior angle of the scapula; and calf—a vertical pinch parallel to the long axis of the leg. The circumference of the arm relaxed (measured at the level of the marked mid-acromiale-radiale), arm flexed (the maximum girth of the right upper arm, which is raised anteriorly to the horizontal with the forearm flexed at 90° to the upper arm, measured perpendicularly to the long axis of the arm) and calf (the maximum girth of the calf at the level of the marked medial calf skinfold site, measured perpendicularly to the long axis of the leg) was measured with anthropometric tape. Moreover, elbow width (measured between the medial and lateral epicondyles of the humerus) and knee width (measured between the medial and lateral epicondyles of the femur) were measured with sliding caliper (GPM, Swiss). For knee measurement, participants were seated on a chair with legs bent at 90˚.

All morphological traits were used to calculate somatotype components of endomorphy, mesomorphy and ectomorphy according to the Heath–Carter anthropometric method [26]. The following equations were used for calculating somatotype:

Endomorphy=-0.7182+0.1451×∑SF–0.00068×∑SF2+0.0000014×∑SF3

where ∑SF = (sum of triceps, subscapular and supraspinale skinfolds) multiplied by (170.18/height in cm).

Mesomorphy=0.858×humerusbreadth+0.601×femurbreadth+0.188×correctedarmgirth+0.161×correctedcalfgirth-height×0.131+4.5.

Three equations were used to calculate ectomorphy according to the height-weight ratio (HWR):

If HWR ≥ 40.75, then ectomorphy = 0.732 × HWR– 28.58.

If HWR is between 38.25 and 40.75, then ectomorphy = 0.463 × HWR– 17.63.

If HWR ≤ 38.25 then ectomorphy = 0.1.

A BIA (Tanita MC-780MA analyzer, Japan), which is a method commonly used in field surveys and as a supplement to conventional anthropometry [27], was used to calculate bilateral body fat and muscle mass (in percentage) based on the directions and procedures of the manufacturer [28]. Participants were instructed to refrain from exercising and eating or drinking anything (other than water) for 3 hours prior to testing, and to void their bladders in order to ensure that test results were not influenced by body temperature, breathing rate, and/or presence of food/beverages in the gastrointestinal tract [29]. During the test, participants stood erect with their bare feet on the contact electrodes while holding the electrodes of the BIA unit in their hands. Participants received a printed copy of their body composition results. All assessments were completed during a single test visit.

When evaluating the degree of asymmetry, it is more beneficial to use symmetry indices rather than mean values of features [14, 15]. Because segmental body composition analysis is adequate for determining the asymmetry levels of main compartments [30], the results of fat mass (%) and muscle mass (%) for both upper and lower limbs were taken from the BIA assessment. In the present study, the dominance of either right or left sides was not important; rather, the levels of asymmetry for the right and left sides were evaluated. That is why the absolute asymmetry index was used for comparisons between them, according to the following equation [31]:

$$\text{AA}=\left|{\text{x}}_{r}-{\text{x}}_{\text{l}}\right|\text{/}\left(\left({\text{x}}_{r}{+\text{x}}_{\text{l}}\right)/2\right)\times 100,,$$

where:

x_r_ = the value of a given characteristic determined on the right side [%]

x_l_ = the value of a given characteristic determined on the left side [%]

A value of zero for this index indicates that the variables x_r_ and x_l_ are the same in right and left extremities, and that there is perfect symmetry in particular morphological parameters (fat mass and muscle mass) between them.

### Statistical analyses

All parameters were reported as mean values ± standard deviation. The normal Gaussian distribution and homoscedasticity of the data were tested by the Shapiro–Wilk and Levene’s tests, respectively. To explore possible differences between sexes for all parameters (arm fat mass, fat mass of legs, muscle mass of arms, and muscle mass of legs), descriptive characteristics were compared using Student’s t-tests for continuous variables and the Mann–Whitney U test for continuous non-normally distributed variables.

To describe differences between groups, effect sizes were calculated as the difference between means divided by the pooled SD. Using Cohen’s [32] criteria, an effect size ≥ 0.20 and < 0.50 was considered small, with ≥ 0.50 and < 0.80 being considered medium, and ≥ 0.80 considered large.

Differences between body types (ectomorph, endomorph and mesomorph) were tested using one-way analysis of variance (ANOVA), while the Tukey post-hoc procedure was used to identify specific differences.

In the absence of interactions, only the main effects were analyzed. This analysis was the basis for creating models of multifactorial ANOVA for each isokinetic variable when significant interactions were present.

A 2 × 3 (sex × body type) repeated-measures ANOVA was used for the AA index of the fat mass (%) and muscle mass (%) of arms and legs. A value of p < 0.05 was considered statistically significant. All analyses were performed using Statistica 13.3 (TIBCO Software Inc., 2017).

## Results

**Fig 1:**
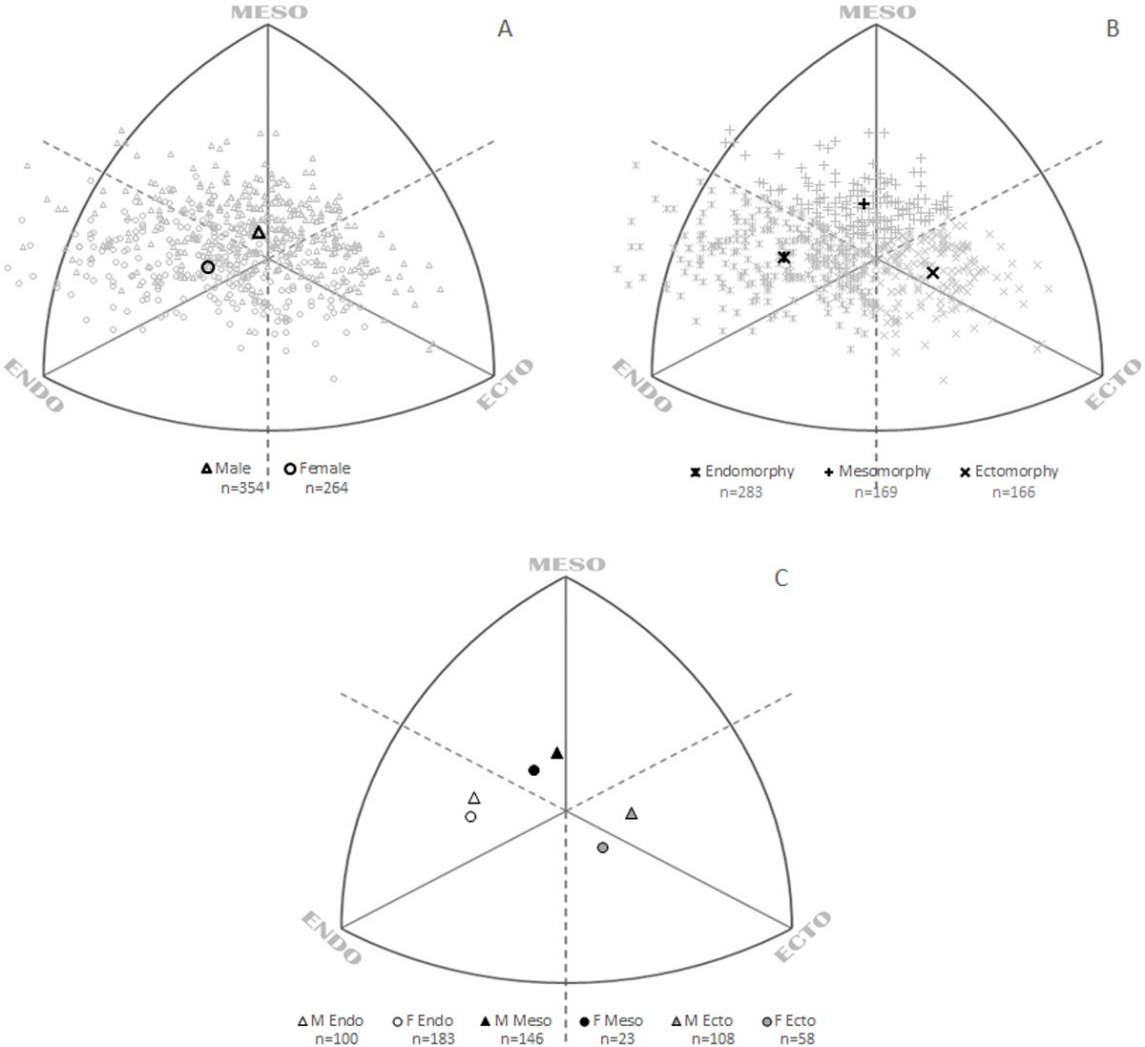
Distribution of students’ somatopoints. A. Groups according to sex, with the mean somatotype for males and females. B. Groups according to components, with the mean somatotype for endomorphs, mesomorphs and ectomorphs. C. Mean somatotypes for groups according to sex (M = male, F = female) and components (Endo = endomorphy, Meso = mesomorphy, Ecto = ectomorphy).

The total sample (n = 618) was divided into three groups according to the predominance of one component (endomorph, mesomorph or ectomorph) using the Heath–Carter method (Fig 1). The endomorph group included 100 males and 183 females, the mesomorph group 146 males and 23 females, and the ectomorph group 108 males and 58 females (Fig 1C).

The sex-specific descriptive statistics of the total sample are summarized in Table 1.

**Table 1:** Morphological characteristics (mean ± SD) of youth according to sex.

Parameters	Male		Female		p-value	d	Effect size
Body height (cm)^a^	180.64	± 5.95	165.92	± 6.27	≤0.0001	2.41	large
Body mass (kg)^b^	75.95	± 9.83	60.53	± 8.74	≤0.0001	1.66	large
BMI (kg/m^2^)^b^	23.26	± 2.50	21.93	± 2.70	≤0.0001	0.51	medium
Fat mass (%)^b^	14.36	± 4.64	24.58	± 5.88	≤0.0001	1.93	large
Muscle mass (%)^b^	61.91	± 6.54	43.16	± 4.80	≤0.0001	3.27	large
Endomorphy^b^	3.23	± 1.16	4.29	± 1.13	≤0.0001	0.92	large
Mesomorphy^a^	3.46	± 0.58	2.89	± 0.53	≤0.0001	1.04	large
Ectomorphy^b^	2.98	± 1.17	2.70	± 1.29	0.0125	0.22	small

p ≤ 0.05 = significant difference

^a^ Student’s t-test

^b^ Mann–Whitney U test

SD = standard deviation

Statistically significant differentiation was observed for all analyzed morphological parameters. The greatest differences were observed for body height, body mass, fat mass, muscle mass, endomorphy and mesomorphy; an average difference in BMI was observed; and the smallest difference was noted for ectomorphy (Table 1).

**Fig 2:**
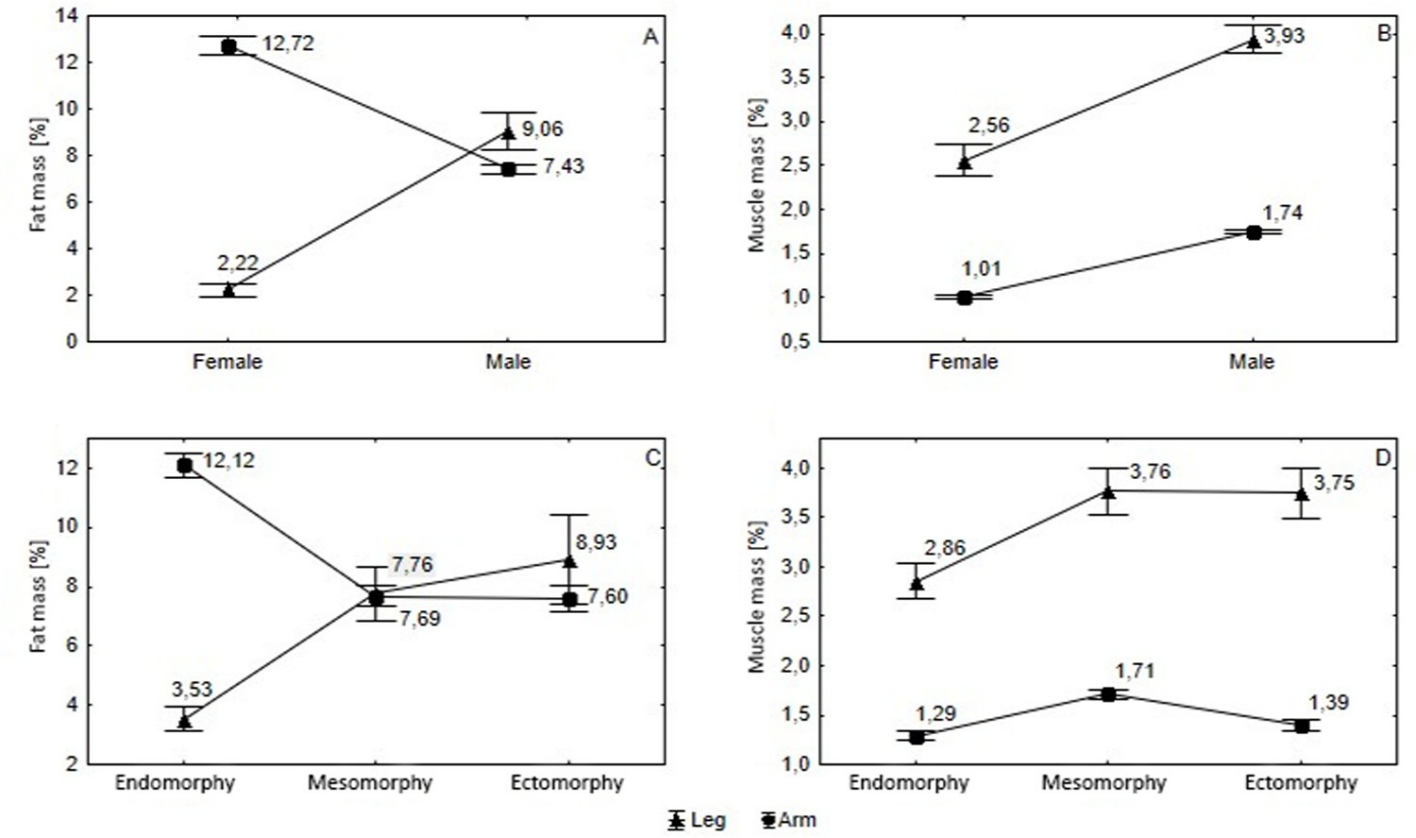
Absolute asymmetry index of basic somatic characteristics according to sex (A, B) and somatotype (C, D) for legs and arms.

Absolute asymmetry index values of basic somatic characteristics according to sex and somatotype for legs and arms are presented in Fig 2.

Significant differences in %AA were observed between males and females in all cases (p ≤ 0.0001). In %AA (fat), there was a statistically significant difference between sexes in the upper (p ≤ 0.0001, d = 1.91) and lower limbs (p ≤ 0.0001, d = 1.20), with higher values observed for arms (Fig 2A).

Similar relationships were demonstrated in %AA (muscle) in the upper and lower limbs (p ≤ 0.0001, d = 3.50; p ≤ 0.0001, d = 0.92, respectively). However, larger differences were recorded in arms (Fig 2B).

When considering body type, the greatest variation in upper-limb fat asymmetry was observed in the endomorphic body type compared to the mesomorphic (p ≤ 0.0001, d = 1.51) and ectomorphic (p ≤ 0.0001, d = 0.76; Fig 2C) types. Moreover, the asymmetry of lower-limb fat was least in the endomorphic type when compared to mesomorphic (p ≤ 0.0001, d = 0.84) and ectomorphic (p ≤ 0.0001, d = 0.72; Fig 2D) types.

Considering the muscularity of upper limbs by somatotype, asymmetry was greatest in the mesomorphic group compared to the endomorphic (p ≤ 0.0001, d = 1.11) and ectomorphic (p ≤ 0.0001, d = 0.88) groups. In the lower limbs, the smallest difference in muscularity was demonstrated in individuals with the endomorphic body type. The interactions between somatic characteristics, sex and body type are presented in Table 2.

**Table 2:** Descriptive characteristics of participants according to sex and body types.

Parameters	Sex	Endomorphy	Mesomorphy	Ectomorphy
Height (cm)	M	181.71 ± 6.18	178.97 ± 5.85	181.88 ± 5.35
	F	165.64 ± 5.82	162.27 ± 5.34	168.20 ± 7.15
Body mass (kg)	M	82.69 ± 9.62^a^^,^^c^	76.91 ± 8.17^a^^,^^b^	68.42 ± 6.44^a^
	F	62.78 ± 8.32^d^	58.98 ± 7.09	54.07 ± 7.20
BMI (kg/m^2^)	M	24.99 ± 2.25	23.95 ± 1.79	20.72 ± 1.24
	F	22.80 ± 2.49	22.32 ± 1.77	19.03 ± 1.23
Fat mass (%)	M	18.39 ± 3.86	13.82 ± 3.73	11.35 ± 3.69
	F	26.50 ± 5.20	20.65 ± 4.87	20.08 ± 5.00
Muscle mass (%)	M	64.22 ± 6.08^a^^,^^d^	63.26 ± 6.28^a^^,^^b^	57.95 ± 5.47^a^
	F	43.60 ± 4.51	44.40 ± 4.57	41.28 ± 5.31

^a ^Significantly higher than in females (endomorphy: p ≤ 0.0001, d = 1.11; mesomorphy: p ≤ 0.0001, d = 2.34; ectomorphy: p ≤ 0.0001, d = 2.10).

^b^ Significantly higher than ectomorphy (body weight: p ≤ 0.0001, d = 1.15; muscle mass: p ≤ 0.0001, d = 0.90).

^c^ Significantly higher than mesomorphy (p ≤ 0.0001, d = 0.65) and ectomorphy (p ≤ 0.0001, d = 1.74).

^d ^Significantly higher than ectomorphy (p ≤ 0.0001, d = 1.12).

M = Male; F = Female

Statistically significant effects of sex and body type interaction were observed for body mass (p = 0.0037) and for muscle mass (p = 0.0021; Table 2). Males of all three body types had significantly higher level of both of those components than endomorphy (p ≤ 0.0001), mesomorphy (p ≤ 0.0001) and ectomorphy (p ≤ 0.0001) females. However, there were no such interaction with BMI (p = 0.3525) or fat mass (p = 0.2975).

Notably, an interaction between sex and body type in the asymmetry of fat and muscle mass in arms and legs is presented in Table 3.

**Table 3:** Differences in the percentage of absolute asymmetry in fat and muscle mass of arms and legs (%) according to sex and body type.

Parameters	Sex	Endomorphy		Mesomorphy		Ectomorphy	
Arm fat mass	M	9.02	± 1.92^a^	7.09	± 1.47	6.42	± 1.68
	F	13.81	± 2.98^a^^,^^b^	11.47	± 2.35^b^	9.79	± 3.01^b^
Leg fat mass	M	6.38	± 4.17^d^	8.62	± 6.12^d^	12.12	± 10.74^c^^,^^d^
	F	1.97	± 1.67	2.26	± 2.64	2.99	± 3.31
Arm muscle mass	M	1.78	± 0.23	1.81	± 0.25	1.62	± 0.22
	F	1.02	± 0.15	1.06	± 0.17	0.96	± 0.18
Leg muscle mass	M	3.59	± 1.58	3.88	± 1.42	4.32	± 1.44
	F	2.46	± 1.38	3.05	± 1.96	2.68	± 1.59

^a^ Significantly higher than in the ectomorphic (male: p ≤ 0.0001, d = 1.44; female: p ≤ 0.0001, d = 1.34) and mesomorphic (male: p ≤ 0.0001, d = 1.13; female: p = 0.0073, d = 0.87) groups.

^b^ Significantly higher than in males (mesomorphic: p ≤ 0.0001, d = 2.23; endomorphic: p ≤ 0.0001, d = 1.91; ectomorphic: p ≤ 0.0001, d = 1.38).

^c^ Significantly higher than in endomorphic (p ≤ 0.0001, d = 0.70) and mesomorphic (p = 0.0002, d = 0.40) male groups.

^d^ Significantly higher than in females (ectomorphic: p ≤ 0.0001, d = 1.15; endomorphic: p ≤ 0.0001, d = 1.39; mesomorphic: p = 0.0029, d = 1.35).

M = Male; F = Female

The data highlights interactions between sex and body type for the asymmetry of fat mass in the arms and legs (Table 3). Endomorphic males and females had significantly greater arm fat mass asymmetry than did ectomorphic males (p ≤ 0.0001) and females (p ≤ 0.0001), as well as the mesomorphic male (p ≤ 0.0001) and mesomorphic female (p = 00.73) groups. Additionally, however, women in all three body types had significantly greater asymmetry levels of arm fat mass than mesomorphic (p ≤ 0.0001), endomorphic (p ≤ 0.0001) and ectomorphic (p ≤ 0.0001) males.

Significantly differences in fat mass asymmetry between sex also concerned legs. In this case endomorphic male had higher level of asymmetry than in ectomorphic (p ≤ 0.0001), endomorphic (p ≤ 0.0001) and mesomorphic (p = 0.0029) females. Ectomorphic males also had significantly higher differences than did endomorphic (p ≤ 0.0001) and mesomorphic (p = 0.0002) males.

No large differences were observed in arm and leg muscle mass asymmetry between sex and body types.

## Discussion

The purpose of the study was to determine the level of morphological asymmetry in the general population of Polish youth with regard to sex and body type. Our results show that certain body types seemed to be associated with specific distributions of fat and muscle tissue. Present findings clearly indicate that the greatest asymmetry in arm-fat value were observed among students characterized by the endomorphic body type. On the other hand, the smallest side-to-side variations in fat were observed in the legs of endomorphic individuals. In the case of muscle mass, the greatest variations were observed in the arms of mesomorphic students, while the smallest variations were observed in the legs of endomorphic students. The results obtained that indicated greater asymmetry levels between upper limbs compared to those of lower limbs, regardless of the analyzed morphological trait, could be explained by the upper extremities being more engaged in different unilateral daily tasks in comparison to lower extremities (especially bilateral movements such as walking or running) [5, 33]. Moreover, we indicated that in general, the fatter the individual, the greater the asymmetry in fat mass in their upper limbs—and similarly, the more muscular the individual, the greater the asymmetry in muscle mass in their upper limbs.

In the present study, somatotype components were compared between sexes. The classification of males was predominantly mesomorphic with a secondary endomorphic component, while females were primarily endomorphic with a secondary mesomorphic component. Other studies have found similar results [34–37], though asymmetry level was not considered.

Therefore, the interaction was identified between sex and body type in the asymmetry level of both arm and leg fat mass. Ozener [6] found that males with a lower- or higher-than-average percentage of fat mass tend to be more asymmetric, whereas Manning [23] observed, that females with less body fat have more symmetrical structures. It was stated, that larger traits may have a greater likelihood of becoming asymmetric [38]. Our investigation yields a similar conclusion. In our study arm fat mass asymmetry for the mesomorphic and ectomorphic body types among females was significantly higher than among males in our research. Leg fat mass asymmetry in all body types among males was significantly higher than in females, but significantly higher in ectomorphic than in endomorphic and mesomorphic individuals, and significantly higher in males than in females.

The present study provides new data on the morphological asymmetry of body composition in a general population of Polish youth, according to somatotype and sex. Males and females with the highest levels of body fat (endomorphic body type) exhibited the most exaggerated asymmetry of this component in the arms, whereas the greatest differentiation between the legs was mostly observed among ectomorphic body types. In the case of muscle mass, the greatest side-to-side arm differences were observed among mesomorphic males and females as well; however, in the case of legs, the greatest differences in muscle mass were observed among ectomorphic males and mesomorphic females.

As previously indicated, certain negative medical consequences may be attributable to morphological asymmetry, which may also be linked to functional mobility. It follows, then, that the assessment of asymmetry levels is especially important in a prophylactic context, wherein it may be used to identify and minimize threats to health and mobility. In the case of the general population, the information is limited, as it is also among students, who are typically about 19 years old, which is an age when general growth has ended, but substantial physical decline has not yet begun. It seems that inter-limb morphological asymmetry should be monitored at every stage of ontogeny to observe and indicate its connection with physical performance, daily functioning and health consequences such as back pain, loss of balance or gait ability. Therefore, the maintenance and deepening of morphological asymmetry during the entire lifespan could lead to health problems in later life. Due to limited data on the relationship between inter-limb fat and muscle mass asymmetry with functional asymmetry among youth who are not engaged in organized sports activities, it seems important to study such association in future research.

## Supporting information

S1 Database (XLSX)
